# A Network Analysis of Smartphone Addiction, Depression, Anxiety, Fatigue, Sleep, and Learning Engagement in Nursing Students: A Cross-Sectional Study

**DOI:** 10.3390/healthcare14121686

**Published:** 2026-06-12

**Authors:** Dain Jeong, Youngsil Lee

**Affiliations:** 1 Department of Nursing, Changshin University, Changwon 51352, Republic of Korea; dainj@cs.ac.kr; 2 Department of Nursing, Masan University, Changwon 51217, Republic of Korea

**Keywords:** nursing students, learning engagement, sleep quality, smartphone addiction, network analysis

## Abstract

**Highlights:**

**What are the main findings?**

- Network analysis was used to examine interrelationships among smartphone addiction, depression, anxiety, fatigue, sleep quality, and learning engagement in nursing students.
- Strong associations were observed between fatigue and depression, and depression emerged as a central node within the estimated network.

**What are the implications of the main findings?**

- Learning engagement was embedded within the interconnected psychological and behavioral network of nursing students.
- The findings highlight conditional associations among psychological and behavioral factors rather than causal or directional relationships.

**Abstract:**

**Objectives**: This study used psychological network analysis to examine the interrelationships among smartphone addiction, depression, anxiety, fatigue, sleep quality, and learning engagement in nursing students. **Methods**: A cross-sectional survey was conducted among 200 nursing students in South Korea using validated self-report instruments. Psychological network analysis was performed using the qgraph and bootnet packages in R. A non-regularized partial correlation network based on Spearman correlations was estimated, and bootstrap was conducted to evaluate the stability and accuracy of network estimates. **Results**: The strongest positive association was observed between fatigue and depression, whereas smartphone addiction showed the strongest negative association with learning engagement. Depression demonstrated relatively higher centrality within the network, while anxiety showed comparatively lower centrality values. Strength and expected influence estimates demonstrated acceptable stability. **Conclusions**: The findings suggest meaningful associations among depression, fatigue, sleep quality, smartphone addiction, and learning engagement in nursing students. Learning engagement demonstrated relatively strong connectivity within the network, highlighting its close association with psychological and behavioral factors. These findings support the utility of network analysis for understanding complex interrelationships among psychological variables in nursing students.

## 1. Introduction

Nursing students often face unique academic and emotional demands, making their psychological well-being critical to their learning success [1]. Among the various psychological constructs relevant to learning engagement, smartphone addiction, anxiety, depression [2], fatigue [3], and sleep quality [4] have emerged as key factors that may hinder learning engagement. In particular, learning engagement, defined as students’ active cognitive, emotional, behavioral, and participatory involvement in educational activities, can be negatively affected by psychological distress and behavioral dysregulation [5].

The widespread use of smartphones has transformed student life, offering convenience but also posing risks [6]. Excessive or problematic smartphone use―termed smartphone addiction―has been linked to a variety of adverse outcomes, including heightened levels of depression, anxiety, fatigue, poor sleep quality, and decreased learning engagement [7,8]. Nursing students may be especially vulnerable due to the dual stressors of demanding coursework and emotionally intense clinical training [9].

These psychological and behavioral challenges tend to co-occur and form dynamic, interrelated systems [10,11]. For example, poor sleep quality may mediate the relationship between smartphone addiction and reduced learning engagement, while fatigue and depressive symptoms further complicate these dynamics [12]. Moreover, downstream effects such as academic burnout and reduced professional self-efficacy have been identified [13].

Traditional linear approaches often treat these variables as separate predictors or outcomes, thereby missing the complex interconnectivity of symptoms [10]. In contrast, network analysis provides a promising methodology by modeling symptoms and behaviors as nodes within a system, with direct associations (edges) revealing potential central or bridging variables [14]. Identifying strongly connected variables within these networks may help clarify the interconnected structure of psychological and behavioral factors among nursing students.

Recent studies have applied network analysis to explore the structure of depression and anxiety [15], the mediating role of fatigue in academic outcomes [16], and the cascading effects of poor sleep quality on smartphone-related dysfunctions [17]. Although previous studies have separately examined smartphone addiction, mental health symptoms, sleep problems, and learning engagement among university students, few studies have simultaneously modeled these variables within a single psychological network in nursing students. Moreover, limited research has applied regularized partial correlation network analysis with stability and accuracy assessment procedures to identify potentially important nodes among these interconnected psychological and behavioral factors. Based on previous literature [2,18], depression and fatigue were expected to demonstrate relatively strong connections with other variables, whereas smartphone addiction was expected to show negative associations with learning engagement.

Given the exploratory nature of psychological network analysis, this study aimed to examine the conditional association structure among smartphone addiction, depression, anxiety, fatigue, sleep quality, and learning engagement in nursing students and to identify central features within the estimated network.

## 2. Methods

### 2.1. Study Design

This study adopted a cross-sectional, correlational design to investigate the network structure of key psychological and behavioral variables related to learning engagement among undergraduate nursing students. Using a network analysis approach, the study aimed to identify central and bridge nodes among smartphone addiction, depression, anxiety, fatigue, sleep, and learning engagement.

### 2.2. Participants and Data Collection

This study aimed to investigate the relationships among smartphone addiction, depression, anxiety, fatigue, sleep, and learning engagement in nursing students. The participants were undergraduate nursing students enrolled in Gyeongsangnam-do, South Korea.

Participants were recruited using a convenience sampling method. Inclusion criteria were: (1) currently enrolled as a full-time nursing student, (2) ownership of a personal smartphone, and (3) willingness to voluntarily participate with informed consent. Students who had a clinically diagnosed psychiatric disorder based on self-reported medical history or were currently taking psychotropic medication were excluded to minimize confounding variables. Consistent with prior network analysis practices, sample size considerations were guided by the stability of network estimation [19]. A previous methodological study [19] suggested that samples exceeding approximately 200 participants allow for more stable network estimation and reliable assessment of centrality indices. Accordingly, a sample of 200 participants was considered adequate for this study.

Data were collected between 1 May and 20 May 2024 using a structured, self-administered questionnaire. The survey was distributed both online via Google Forms and in person, depending on participants’ preferences. The questionnaire consisted of validated instruments assessing smartphone addiction, depression, anxiety, fatigue, sleep quality, and learning engagement, as well as demographic characteristics including age, gender, and academic year. Participation was entirely voluntary, and participants were informed that they could withdraw from the study at any time without any academic disadvantage. The survey required approximately 20 min to complete. A total of 200 questionnaires were collected, and no responses were excluded due to incomplete or careless answering; therefore, all 200 questionnaires were included in the final analysis.

### 2.3. Questionnaire and Measurements

The questionnaire consisted of the following sections: (1) general characteristics of the participants; (2) reason for choosing nursing major; (3) Satisfaction with major and school; (4) Current residence status.

#### 2.3.1. Smartphone Addiction

Smartphone addiction was assessed with the Smartphone Addiction Proneness Scale (SAPS) for adults, developed by the National Information Society Agency [20] in Korea. The scale comprises 15 items categorized into four subdomains: disturbance of daily functioning (5 items), virtual world orientation (2 items), withdrawal (4 items), and tolerance (4 items). Responses are rated on a 4-point Likert scale, with higher scores indicating greater addiction risk. The original study reported a Cronbach’s α of 0.86, whereas it was 0.90 in the current study.

#### 2.3.2. Depression

To evaluate depressive symptoms, the study adopted the Korean version of the Center for Epidemiological Studies Depression Scale (CES-D), originally developed by Radloff [21] and adapted by Cho and Kim [22]. This scale contains 20 items, each rated on a 4-point Likert scale, reflecting symptom frequency over the past week. The Korean version of the CES-D [22] showed a Cronbach’s α of 0.89, and Cronbach’s α was 0.87 in this study.

#### 2.3.3. Anxiety

Anxiety was gauged via the State-Trait Anxiety Inventory (STAI), originally developed by Spielberger et al. [23] and later translated and adapted for Korean populations by Kim and Shin [24]. The state anxiety consists of 20 items measuring state anxiety, each scored on a 4-point Likert scale. The Cronbach’s α in this study was 0.78.

#### 2.3.4. Fatigue

Fatigue levels were measured through the Fatigue Severity Scale developed by Krupp et al. [25] and translated into Korean by Lee et al. [26]. It includes 9 items, each scored on a 7-point Likert scale ranging from 1 (strongly disagree) to 7 (strongly agree), with higher scores representing greater fatigue severity. Lee et al. [26] reported Cronbach’s α of 0.93; Cronbach’s α in this study was 0.88.

#### 2.3.5. Sleep

Sleep was evaluated using the Pittsburgh Sleep Quality Index (PSQI), originally developed by Buysse et al. [27] and later translated and validated into Korean by Sohn et al. [28]. The PSQI consists of 19 self-rated items that assess various dimensions of sleep over the past month, including sleep latency, duration, efficiency, disturbances, and daytime dysfunction. A global score is calculated, with higher scores indicating poorer sleep quality. Sohn et al. [28] reported Cronbach’s α of 0.84; Cronbach’s α in this study was 0.81.

#### 2.3.6. Learning Engagement

Learning engagement was examined using the Student Course Engagement Questionnaire developed by Handelsman et al. [29]. The original instrument consists of 23 items across four domains: skills, emotional, participation, and performance engagement. In this study, the instrument was translated into Korean by the researcher, and its content validity was reviewed by three experts in education and nursing education.

During the expert review process, the three items belonging to the performance engagement subscale were identified as having relatively low content validity because they primarily focused on grade- or score-related aspects, which were considered less conceptually appropriate in the context of Korean nursing students. Accordingly, these items were removed following expert discussion, resulting in a final scale of 20 items. The remaining 20 items demonstrated acceptable content validity, with all item-level content validity index (I-CVI) values of 1.0. These values reflected independent ratings from three experts, all of whom evaluated each retained item as relevant.

Each item was rated on a 5-point Likert scale, with higher scores indicating greater learning engagement. The original instrument demonstrated internal consistency, with Cronbach’s α ranging from 0.76 to 0.82 across its subscales [28]. In this study, Cronbach’s α was 0.91.

### 2.4. Data Analysis

All statistical analyses were conducted using R software (version 4.3.1). Descriptive statistics, including means, standard deviations, frequencies, and percentages, were calculated to summarize participants’ demographic characteristics and main study variables.

The primary analytical approach of this study was psychological network analysis. Because the study variables were derived from multi-item Likert-type instruments and demonstrated non-normal distributions, a non-regularized partial correlation network based on Spearman correlations was estimated. Summed total scores for each construct were treated as approximately continuous variables, consistent with common practice in psychological network analysis involving multi-item scales.

Network estimation was conducted using the qgraph and bootnet packages in R. A non-regularized partial correlation network was estimated using the pcor method implemented in the estimateNetwork function from the bootnet package. Spearman rank correlations were used to compute the correlation matrix, with tied observations handled using average ranks in R. No additional data transformations were applied prior to network estimation. In the estimated network, nodes represented the study variables and edges represented partial correlation coefficients after controlling for all other variables in the network. Edge thickness reflected the strength of associations, with positive edges shown in green and negative edges shown in red.

To evaluate the accuracy and stability of the network, nonparametric bootstrap procedures were conducted using the bootnet package. Edge-weight accuracy was assessed using nonparametric bootstrap confidence intervals, and case-dropping bootstrap procedures were used to evaluate the stability of centrality indices using the correlation stability coefficient (CS-coefficient). Node centrality was primarily assessed using strength and expected influence, which quantify the relative importance of nodes by considering the magnitude and direction of their network connections [30].

### 2.5. Ethical Considerations

This study was conducted in accordance with the ethical principles outlined in the Declaration of Helsinki. Prior to data collection, ethical approval was obtained from the Institutional Review Board of Gyeongsang National University (GIRB-A24-NY-0031). All participants were informed about the purpose, procedures, and voluntary nature of the study. Informed consent was obtained electronically prior to participation. Participants were assured that their responses would remain anonymous and confidential, and that all data would be used exclusively for research purposes. Participants were also informed that they could withdraw from the study at any point without any academic or personal consequences. All collected data were stored in password-protected files and accessible only to the research team. No identifying information was recorded.

## 3. Results

### 3.1. Participant Characteristics and Differences in Learning Engagement by General Characteristics

A total of 200 nursing students participated in the study (82.0% female, 18.0% male). The majority (53.0%) were aged 21–24 years. By academic year, 39.5% were in their second year, followed by fourth (31.5%) and third (29.0%) years. Learning engagement differed significantly according to academic year (F = 4.27, *p* = 0.015). Post hoc Scheffé tests indicated that fourth-year students showed significantly higher learning engagement than second-year students.

Regarding the reason for choosing nursing, 38.5% cited employment prospects; however, learning engagement did not significantly differ by reason for choosing the nursing major (*p* = 0.518). Most students were satisfied (38.5%) or neutral (36.5%) with their major, and learning engagement did not significantly differ according to major satisfaction (*p* = 0.304), although students who reported being very satisfied showed relatively higher scores. Similarly, satisfaction with the school was not significantly associated with learning engagement (*p* = 0.226). Most students lived at home (61.5%), and residence type was not significantly associated with learning engagement (*p* = 0.624) (Table 1).

### 3.2. Levels of Smartphone Addiction, Depression, Anxiety, Fatigue, Sleep and Learning Engagement of Nursing Students

The descriptive statistics of the study variables are presented in Table 2. The mean scores were 33.10 (SD = 7.65) for smartphone addiction, 9.53 (SD = 6.97) for depression, 47.05 (SD = 6.54) for anxiety, 35.42 (SD = 10.30) for fatigue, 7.80 (SD = 2.97) for sleep, and 66.25 (SD = 11.61) for learning engagement. The skewness and kurtosis values ranged from −1.10 to 0.97 and −0.27 to 2.68, respectively, indicating that the distributions of the variables were within acceptable limits for normality (Table 2).

### 3.3. Network Structure

**Figure 1:**
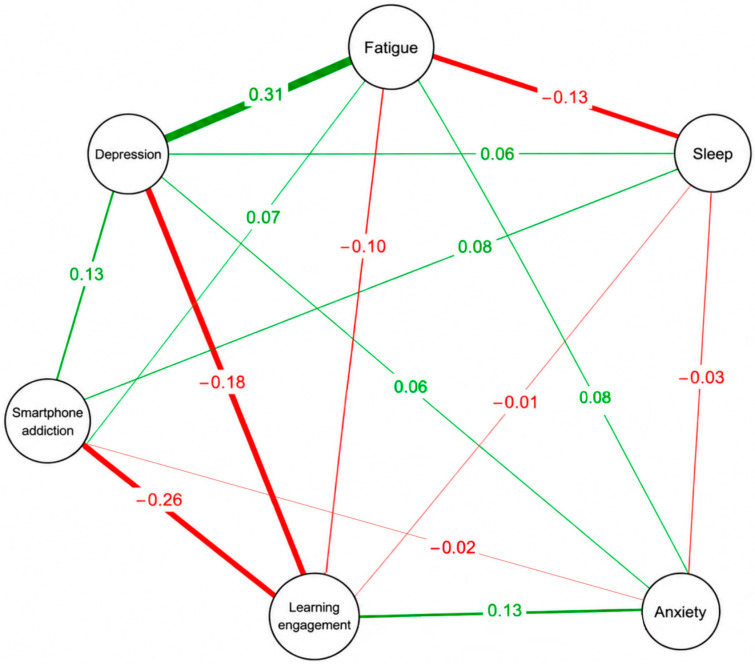
Network structure of partial correlations among smartphone addiction, depression, anxiety, fatigue, sleep quality, and learning engagement. Green edges indicate positive associations, and red edges indicate negative associations.

The estimated network structure revealed several noteworthy associations among the study variables. The strongest positive edge was observed between fatigue and depression (r = 0.31), indicating that participants reporting higher fatigue also tended to report higher depressive symptoms. Conversely, the strongest negative edge was found between smartphone addiction and learning engagement (r = −0.26), suggesting that smartphone addiction may interfere with students’ ability to engage in learning. Additional moderate associations were observed between learning engagement and depression (r = −0.18). A negative association was observed between sleep and fatigue (r = −0.13). Anxiety showed relatively weak connections with other nodes, suggesting a more peripheral position within the network (Figure 1).

### 3.4. Centrality Indices

**Figure 2:**
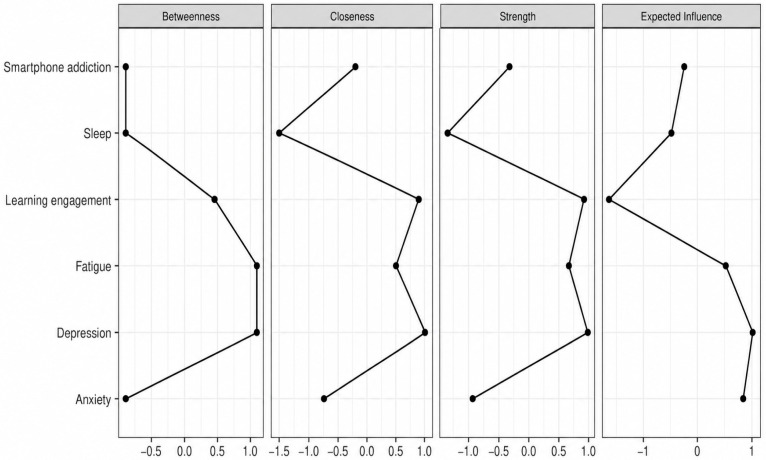
Centrality indices of the estimated network.

Figure 2 presents the centrality indices of the estimated network. Depression showed the highest strength centrality, indicating that it had the strongest overall connections with other variables in the network. Learning engagement and fatigue also demonstrated relatively high strength values, suggesting that these variables played important roles in the network structure. In contrast, sleep and anxiety showed relatively low strength centrality, indicating more peripheral positions within the network. Expected influence showed a similar pattern, with depression demonstrating the highest positive influence in the network (Figure 2).

### 3.5. Stability and Accuracy of Estimates

**Figure 3:**
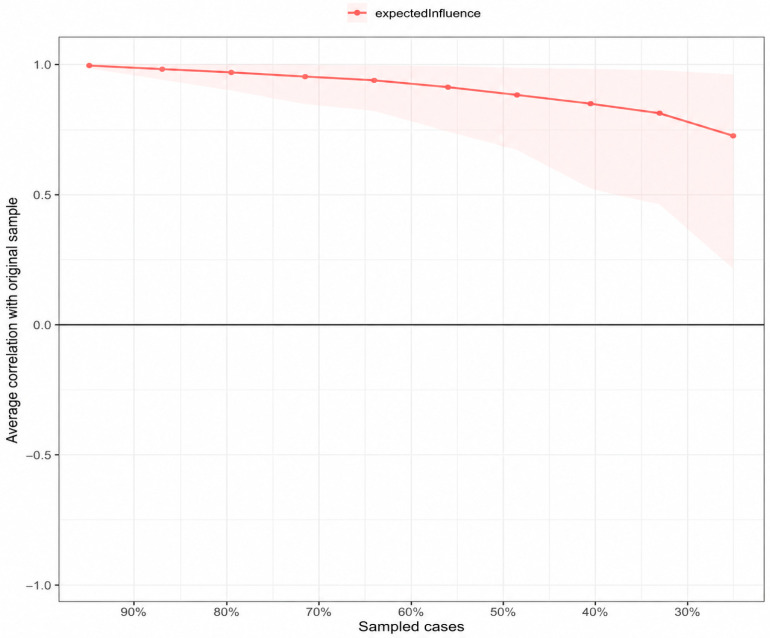
Case-dropping bootstrap results for expected influence stability. The *x*-axis represents the proportion of retained cases, and the *y*-axis represents the average correlation between expected influence estimates from the original network and those from networks re-estimated after dropping increasing proportions of cases. The solid line represents the average correlation, and the shaded area indicates the 95% confidence interval.

We further examined expected influence, which accounts for both positive and negative connections in the network. Depression showed the highest expected influence, indicating that it had the strongest overall impact on other variables in the network. In contrast, learning engagement showed a negative expected influence, reflecting its inverse associations with several nodes. Case-dropping bootstrap analyses indicated that expected influence demonstrated good stability (CS = 0.52), supporting reliable interpretation of the centrality estimates (Figure 3). Detailed edge-weight tables and bootstrapped confidence interval plots for edge-weight accuracy are provided in the Supplementary Materials (Supplementary Table S1, Supplementary Figure S1).

## 4. Discussion

This study utilized network analysis to explore the interrelationships among smartphone addiction, depression, anxiety, fatigue, sleep, and learning engagement in nursing students.

The strongest positive edge was between fatigue and depression, indicating a close link between these two symptoms. This finding reflects the close interrelationship between fatigue and depression, consistent with the high comorbidity reported in previous research [16,31]. A strong negative edge was observed between smartphone addiction and learning engagement, suggesting that overuse of smartphones may hinder students’ academic engagement. This aligns with previous findings that smartphone overuse impairs attention, reduces learning efficiency, and increases academic procrastination [2,4].

In terms of centrality, depression showed the highest values in both strength and expected influence, suggesting that it functions as a key hub influencing multiple other symptoms and behaviors in the network. Learning engagement and fatigue also demonstrated relatively high centrality values, indicating their important roles within the network structure. Conversely, the relatively peripheral role of anxiety in the present network may appear inconsistent with previous studies reporting strong associations among anxiety, depression, and smartphone addiction. One possible explanation is that anxiety shares substantial variance with depression, such that its unique associations become weaker after controlling for other variables in the network. In addition, because network edges represent conditional associations, relationships commonly observed in bivariate analyses may be attenuated when shared variance is considered. Characteristics of the present sample may also have contributed to this pattern. These interpretations remain speculative and should be examined in future research.

The fatigue–depression link is well-documented in mental health research. Fatigue is often considered a transdiagnostic or bridging symptom across affective disorders and frequently co-occurs with depression in both clinical and non-clinical populations [16,32]. The negative relationship between smartphone addiction and learning engagement is consistent with prior research showing that excessive smartphone use interferes with academic performance, reduces concentration, and increases digital distraction [2,33].

Sleep demonstrated associations with fatigue within the estimated network, although its centrality was lower relative to depression, fatigue, and learning engagement. Previous studies have reported that sleep-related symptoms may interact with psychological and behavioral variables within mental health networks [11,17]. However, the present findings should be interpreted cautiously because the observed edge weights reflect non-regularized partial correlations, representing conditional associations among variables after controlling for all other variables in the network.

These findings offer several implications for intervention and mental health promotion among nursing students. The strong association between fatigue and depression suggests that these variables are closely interconnected within the estimated network [16]. The observed negative association between smartphone addiction and learning engagement highlights the close relationship between digital behavior and academic engagement among nursing students [2]. Additionally, the observed associations involving sleep suggest that sleep-related behaviors may warrant further investigation in future longitudinal and intervention studies [34]. For nursing students, who often face irregular schedules, high academic demands, and stressful clinical training, these findings may provide useful directions for future research. Future studies may examine whether sleep hygiene, digital wellness, and depressive symptoms are associated with changes in mental health and academic functioning among nursing students.

Methodologically, this study highlights the advantages of applying network analysis to mental health and behavioral research. Unlike traditional latent variable approaches that conceptualize symptoms as interchangeable indicators of underlying constructs, network analysis conceptualizes symptoms and behaviors as interconnected entities linked through conditional associations. This perspective allows for the identification of specific nodes that function as hubs or bridges within the network, which might be overlooked by traditional methods [10,14]. In particular, the robust stability of strength and expected influence underscores the utility of network analysis in identifying important nodes within psychological and behavioral networks. These indices revealed that depression, along with learning engagement and fatigue, represented structurally important components within the network. However, because the present study was based on cross-sectional data, the findings should not be interpreted as indicating causal or directional relationships among variables. Future longitudinal and experimental studies are needed to further examine the potential roles of these variables within psychological and behavioral networks.

Cultural factors may also shape the observed network structure. In East Asian contexts, where smartphone penetration is extremely high and academic competition is intense, the disruptive impact of excessive smartphone use on learning and mental health may be particularly pronounced [35,36]. Future cross-cultural network studies are needed to examine whether the centrality patterns observed in this study―particularly the central roles of depression, learning engagement, and fatigue―generalize across different sociocultural contexts.

## 5. Limitations

Several limitations should be acknowledged. First, the sample size may have limited the stability of certain centrality indices (e.g., betweenness and closeness), and therefore, these indices were not interpreted. In addition, participants were recruited using convenience sampling from a single geographic region in South Korea, which may limit the generalizability of the findings to other nursing student populations.

Second, the study relied on self-reported questionnaires, which may be subject to social desirability and recall bias. Future research should employ longitudinal designs to examine how these symptom networks evolve over time.

Third, although the modified Korean version of the learning engagement instrument demonstrated high internal consistency in the present study, formal psychometric validation procedures such as factor analysis were not conducted after modification of the original scale. In addition, because all items from the performance engagement subscale were removed, the learning engagement construct assessed in this study does not fully encompass the original multidimensional framework proposed by Handelsman et al. [29]. Therefore, further validation studies are needed to establish the construct validity and comparability of the modified version in Korean nursing students, and findings related to learning engagement should be interpreted with appropriate caution.

Furthermore, several potentially relevant contextual variables were not included in the present network model. Although academic year showed a significant association with learning engagement, it was not incorporated into the network analysis because the study focused on the relationships among the primary psychological and behavioral constructs. In addition, other contextual factors, such as academic stress, clinical training demands, and socioeconomic factors, were not assessed. The omission of these variables may have influenced the observed network structure and centrality estimates. Future studies should examine the potential role of demographic and contextual factors within more comprehensive network models.

## 6. Conclusions

This study used network analysis to examine the interrelationships among smartphone addiction, depression, anxiety, fatigue, sleep quality, and learning engagement in nursing students. The findings revealed strong associations between fatigue and depression and identified depression as a relatively central node within the estimated network. Learning engagement was embedded within this interconnected structure, suggesting that academic engagement is closely associated with psychological and behavioral factors in nursing students. The present findings highlight potentially important associations among depressive symptoms, fatigue, smartphone addiction, sleep quality, and learning engagement. However, because the estimated network was derived from cross-sectional data, the observed relationships should be interpreted as conditional associations rather than causal or directional effects. Overall, this study demonstrates the utility of network analysis in examining the complexity of psychological and behavioral factors in nursing students and contributes to a broader understanding of the interconnected relationships among these variables.

## Figures and Tables

**Table 1:** Participant Characteristics and Differences in Learning Engagement by General Characteristics (N = 200).

Characteristics	Categories	*n*	%	Learning Engagement			
				Mean ± SD	t or F	η^2^	*p*
Gender	Male	36	18.0	66.58 ± 14.19	0.19		0.850
	Female	164	82.0	66.18 ± 11.01			
Age (yr)	≤21	59	29.5	65.49 ± 10.08	4.23	0.019	0.238
	21–24	106	53.0	66.17 ± 12.99			
	25–29	23	11.5	65.35 ± 7.66			
	≥30	12	6.0	72.42 ± 11.25			
Academic year	2nd ^a^	79	39.5	63.35 ± 12.06	4.27	0.042	0.015
	3rd ^b^	58	29.0	67.74 ± 10.61			(c > a ^†^)
	4th ^c^	63	31.5	68.51 ± 11.31			
Reason for choosing nursing major	Matched my entrance exam score	21	10.5	67.05 ± 12.48	0.76	0.011	0.518
	Suited my aptitude	57	28.5	67.75 ± 10.59			
	Recommendation from others (e.g., parents, teachers)	45	22.5	66.47 ± 13.39			
	High post-graduation employment rate	77	38.5	64.79 ± 11.02			
Satisfaction with major	Very satisfied	44	22.0	68.61 ± 12.34	1.22	0.018	0.304
	Satisfied	77	38.5	66.25 ± 11.31			
	Neutral	73	36.5	65.27 ± 11.62			
	Dissatisfied	6	3.0	60.83 ± 8.09			
Satisfaction with school	Very satisfied	30	15.0	70.00 ± 10.68	1.46	0.022	0.226
	Satisfied	94	47.0	65.66 ± 12.64			
	Neutral	64	32.0	65.03 ± 9.93			
	Dissatisfied	12	6.0	68.00 ± 12.98			
Current residence status	Living at home	123	61.5	66.37 ± 10.86	0.47	0.005	0.624
	Dormitory	58	29.0	65.33 ± 12.57			
	Rented room	19	9.5	68.26 ± 13.49			

SD = standard deviation; yr = year; ^†^ Scheffé test: means with different lowercase letters are significantly different.

**Table 2:** The levels of Smartphone Addiction, Depression, Anxiety, Fatigue, Sleep and Learning Engagement of the Participants (N = 200).

Variables	Mean ± SD	Skewness	Kurtosis
Smartphone addiction	33.10 ± 7.65	0.04	0.01
Depression	9.53 ± 6.97	0.97	1.57
Anxiety	47.05 ± 6.54	−1.10	2.68
Fatigue	35.42 ± 10.30	−0.05	−0.27
Sleep	7.80 ± 2.97	0.09	−0.16
Learning engagement	66.25 ± 11.61	−0.01	0.61

## Data Availability

The data in this study are available from the corresponding author on reasonable request. The data are not publicly available due to privacy and ethical restrictions.

## Supplementary Materials

The following supporting information can be downloaded at: https://www.mdpi.com/article/10.3390/healthcare14121686/s1, Figure S1: Bootstrapped confidence intervals for edge-weight accuracy; Table S1: Edge Weights of the Estimated Partial Correlation Network.
