# Supplementary material for: A Network Analysis of Smartphone Addiction, Depression, Anxiety, Fatigue, Sleep, and Learning Engagement in Nursing Students: A Cross-Sectional Study
Source: Healthcare (Basel). 2026 Jun 12;14(12):1686. doi: 10.3390/healthcare14121686 (PMC13300454; doi:10.3390/healthcare14121686)

Supplementary Table S1. Edge Weights of the Estimated Partial Correlation Network

Node 1	Node 2	Weight
Fatigue	Depression	0.31
Learning engagement	Smartphone addiction	–0.26
Learning engagement	Depression	–0.18
Learning engagement	Anxiety	0.13
Depression	Smartphone addiction	0.13
Sleep	Fatigue	–0.13
Learning engagement	Fatigue	–0.10
Sleep	Smartphone addiction	0.08
Anxiety	Fatigue	0.08
Fatigue	Smartphone addiction	0.07
Sleep	Depression	0.06
Anxiety	Depression	0.06
Sleep	Anxiety	–0.03
Anxiety	Smartphone addiction	–0.02
Learning engagement	Sleep	–0.01

Note: Edge weights represent non-regularized partial correlation coefficients. Values close to zero indicate very weak conditional associations and were not substantively interpreted in the present study. Interpretation focused primarily on the strongest and most stable associations within the network.

Supplementary Figure S1. Bootstrapped confidence intervals for edge-weight accuracy

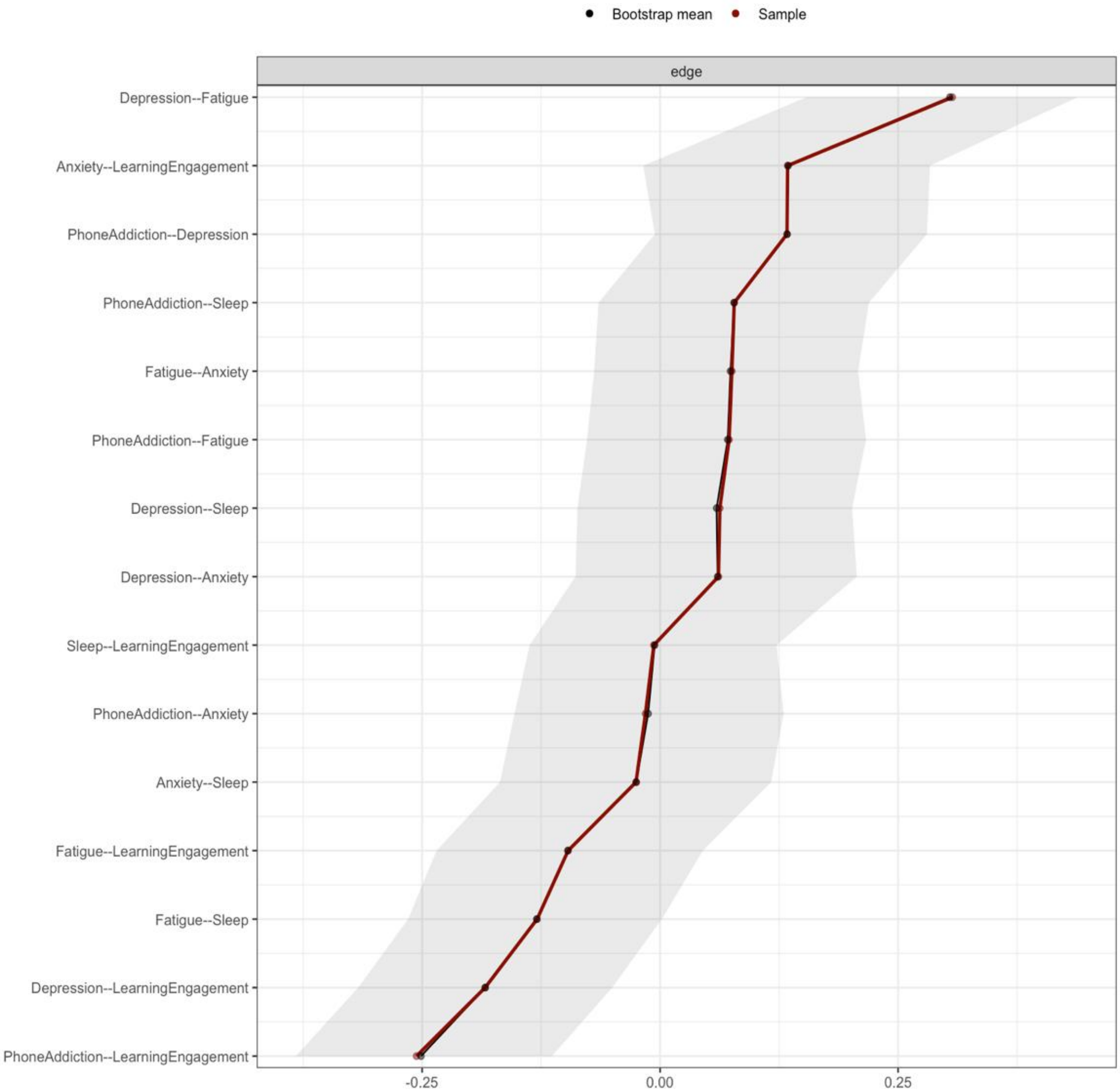

Supplement: Supplementary file 1 [file healthcare-14-01686-s001.zip › healthcare-4221059-supplementary.pdf]
